# Expanding the TBL1XR1 Disease Spectrum: Generalized Dystonia Associated with a New Genetic Variant

**DOI:** 10.5334/tohm.1242

**Published:** 2026-09-03

**Authors:** Maximilian Johannes Löw, Felix Bernsdorff, Christiane Weinrich, Saskia Biskup, Jerome Jüngling, Christoph van Riesen, Fabian Maass

**Affiliations:** 1 Department of Neurology, University Medical Center Göttingen, Göttingen, Germany; 2 CeGaT, Center for Genomics and Transcriptomics, Tübingen, Germany; 3 German Center for Neurodegenerative Diseases (DZNE), Göttingen, Germany

**Keywords:** TBL1XR1, generalized dystonia, Pierpont syndrome

## Abstract

**Background:**

A growing number of identified genes increasingly reveal genetic overlaps between neurodevelopmental disorders and combined dystonia syndromes.

**Case report:**

We report a 61-year-old man with a neurodevelopmental disorder, mild ataxic signs and generalized dystonia who had been misdiagnosed with cerebral palsy for 40 years. Whole-exome sequencing identified a novel heterozygous pathogenic frameshift variant in TBL1XR1.

**Discussion:**

TBL1XR1 variants are classically associated with Pierpont syndrome and autism spectrum disorder. Although movement disorders have been reported, this case suggests generalized dystonia as a possible additional manifestation. It highlights the value of retrospective genetic phenotyping and next-generation sequencing in adults with long-standing neurodevelopmental diagnoses.

TBL1XR1 encodes the WD40 repeat–containing protein TBLR1, a core subunit of the NCoR/SMRT transcriptional repressor complex, which regulates retinoic acid and thyroid hormone-dependent gene expression via HDAC3-mediated histone deacetylation [1]. Pathogenic variants in TBL1XR1 have been associated with two main clinical presentations: Pierpont syndrome (PRPTS, #OMIM 602342) and Intellectual Developmental Disorder, Autosomal Dominant 41 (MRD41, #OMIM 616944), a broader spectrum of neurodevelopmental disorders including autism spectrum disorders and intellectual disability. While TBL1XR1-associated disorders are primarily characterized by neurodevelopmental impairment and early-onset epilepsy, movement disorders are neither frequent nor a defining feature. Chorea, stereotypies and ataxia have been occasionally reported in pediatric patients, whereas generalized dystonia, particularly in adulthood, has not been described [2,3].

A 61-year-old Caucasian male, accompanied by his mother, was referred to our specialized outpatient clinic for movement disorders with an unclassified neurodevelopmental disorder and a progressive hyperkinetic movement disorder. There was no family history of dystonia or other movement or neurodevelopmental disorders. According to his medical history, his birth was uncomplicated (Weight: 2950 g, Length: 54 cm, Head circumference: 34 cm), although minor signs of immaturity were reported (not further defined). From early infancy he showed delayed developmental milestones. He received specialized support from the age of five, attended a special educational needs school and subsequently worked in a workshop for disabled people for several decades. While he could perform skillful work, his intellectual disability and concentration deficits became increasingly evident. Medical records from his late teens mentioned pes cavus with claw toes and marked palmoplantar hyperhidrosis. At the age of 18 cervical dystonia was first noted, which developed over several years into a generalized dystonia including spasmodic dysphonia, oromandibular involvement, and axial as well as appendicular symptoms. In 1981, one year after cervical dystonia was noticed, a genetic consultation yielded a normal 46, XY karyotype. Consequently, his symptoms were misclassified for over forty years as the result of a presumed early childhood brain damage of an unknown origin, despite their progressive course. The patient is now retired and lives in his own apartment. He manages most activities of daily living independently but requires assistance with administrative tasks.

Upon presentation at our movement disorder outpatient clinic at the age of 61, the patient presented with severe generalized dystonia (Video 1). Clinical examination revealed left-sided laterocollis and right-sided torticollis, accompanied by shoulder elevation on the right. Oromandibular dystonia resulted in intermittent jaw-opening patterns, lingual malposition and spasmodic dysphonia. Axial dystonia manifested as variable lateral flexion of the trunk. In addition, there was dystonic posturing of both hands. The patient exhibited mild signs of ataxia, such as light overshooting during finger chase testing and left-sided dysdiadochokinesia. Gait was broad-based accompanied by postural instability. Oculomotor examination revealed saccadic smooth-pursuit eye movements without saccadic hypermetria. Contrary to the information in older records, our current clinical examinations revealed neither claw toes nor a pes cavus. Notably, his head circumference (53.5 cm) was within the normal range and he lacked the typical palmar fat pads or dysmorphic facial features associated with Pierpont syndrome [2]. Cognitive testing yielded an MMSE score of 16/30 points; consequently, further in-depth analysis was not performed.

**Video 1:** **Patient examination.** Segment 1: The patient demonstrates oromandibular dystonia with spasmodic dysphonia and generalized dystonia with right-sided torticollis and left-sided laterocollis. No postural tremor can be detected. Segment 2: Finger-to-nose is normal and finger chase testing shows mild signs of ataxia. Segment 3: Rapid alternating movements reveal moderate dysdiadochokinesia, more prominent on the left side. Segment 4: Finger tapping shows no signs of bradykinesia. Segment 5: Heel-shin slide is slightly abnormal, again more prominent on the left side. Segment 6: Truncal dystonia with tilt to the right is evident. The patient walks with a broad-based gait and is unable to perform tandem gait.

A cranial MRI revealed no evidence of early structural brain damage and was otherwise unremarkable, except for a non-specific, older temporomesial defect. Long-term EEG in 2023, performed prior to the first visit in our clinic to rule out epilepsy or cortical myoclonus, showed diffuse general changes without evidence of epileptiform discharges. Nerve conduction studies revealed mild axonal sensorimotor polyneuropathy. Comprehensive whole-exome sequencing, encompassing SNV/small indel detection and CNV analysis, revealed a heterozygous pathogenic variant in TBL1XR1 (Genome Build: hg19; DNA Level: NM_024665.7:c.1199_1209del; RNA Level: r.1199_1209del; Protein Level: p.(Ser400Thrfs*4)). The variant was identified as part of a validated diagnostic workflow at a sequencing depth of more than 100 reads and was manually verified. This deletion introduces a frameshift and a premature termination codon at position 400 within the highly conserved WD40-repeat region at the C-terminus. This region is essential for protein-protein interactions within the repressor complex and for binding hypoacetylated histones [2]. As the variant affects exon 11 of 14, the mutant transcript is expected to undergo nonsense-mediated mRNA decay, resulting in loss of function. The variant is absent from the gnomAD database and is classified as pathogenic according to ACMG/AMP criteria (PVS1, PM2). The analysis did not reveal pathogenic variants in other dystonia-associated genes, although additional variants of uncertain significance were identified (NKX2-1: c.505G>T; p.(Gly169Cys) and PDE10A: c.1637C>T; p.(Thr546Met). A manual evaluation was conducted for both variants; however, this did not yield robust evidence that the alteration was relevant to the disease. Independent re-analysis within a German reference network for rare neurological diseases confirmed these findings. Parental genetic testing was not performed, since the father passed away and the mother refused to undergo genetic counseling. Pharmacological treatment with trihexyphenidyl provided slight improvement of axial dystonia at a daily dose of 4 mg, however, dose escalation led to confusion and worsening of dysarthria, prompting discontinuation. The current management relies on botulinum toxin injections for cervical and vocal symptoms, alongside multidisciplinary care involving physiotherapy, occupational therapy, and speech therapy. Deep brain stimulation (DBS) has been discussed as a potential treatment option for his generalized dystonia but was deferred due to the patient’s preserved functional status.

The case of our patient is unique because it suggests that a loss-of-function variant resulting in haploinsufficiency likely mediated by nonsense-mediated mRNA decay may be associated with a phenotype distinct from the classic Pierpont syndrome (usually associated with dominant-negative missense mutations), although variants with premature truncation from frameshift leading to Pierpont syndrome have been described [1,2]. Despite the documentation of a small number of loss-of-function variants in gnomAD, the assumption that the disease is driven by a loss-of-function mechanism is supported by the fact that TBL1XR1 is objectively subject to strong selective constraint regarding loss-of-function variations (pLI = 1; o/e = 0.075, LOEUF score). The differential diagnosis of dystonia encompasses genes associated with isolated dystonia (such as e.g. TOR1A, THAP1, and GCH1), as well as a growing number of genes linked to complex neurodevelopmental disorders. KMT2B variants in particular show similarities regarding early developmental delay followed by generalized dystonia [4,5]. Although motor symptoms were present from early childhood and the documentation of claw toes and pes cavus in older medical records might suggest foot dystonia dating back to adolescence, the striking feature of this case is the continuous progression into severe generalized dystonia over more than four decades, resulting in a clinically relevant adult phenotype that may have remained underrecognized due to the scarcity of reported adult TBL1XR1 cases. The identification of a TBL1XR1 variant in this 61-year-old suggests that this gene may also be considered in patients with neurodevelopmental disorder and progressive generalized dystonia, although causality cannot be established based on a single case. Reverse phenotyping after whole exome sequencing were crucial in correcting a forty-year-long misdiagnosis of cerebral palsy, highlighting that TBL1XR1-associated syndromes might not be merely static childhood conditions but may follow a progressive course and present a significantly different phenotype in adulthood.

## References

[1] Mastrototaro G, Zaghi M, Massimino L, et al. TBL1XR1 Ensures Balanced Neural Development Through NCOR Complex-Mediated Regulation of the MAPK Pathway. Front Cell Dev Biol. 2021;9:641410. DOI: 10.3389/fcell.2021.641410 33708771 PMC7940385

[2] Nagy A, Molay F, Hargadon S, et al. The spectrum of neurological presentation in individuals affected by TBL1XR1 gene defects. Orphanet J Rare Dis. 2024;19(1):79. DOI: 10.1186/s13023-024-03083-3 38378692 PMC10880200

[3] Kobayashi Y, Tohyama J, Kato M, et al. High prevalence of genetic alterations in early-onset epileptic encephalopathies associated with infantile movement disorders. Brain Dev. 2016;38(3):285–292. DOI: 10.1016/j.braindev.2015.09.011 26482601

[4] Zech M, Jech R, Boesch S, et al. Monogenic variants in dystonia: an exome-wide sequencing study. Lancet Neurol. 2020;19(11):908–918. DOI: 10.1016/S1474-4422(20)30312-4 33098801 PMC8246240

[5] Brüggemann N. Contemporary functional neuroanatomy and pathophysiology of dystonia. J Neural Transm (Vienna). 2021;128(4):499–508. DOI: 10.1007/s00702-021-02299-y 33486625 PMC8099808

